# Changes in endemic patterns of respiratory syncytial virus infection in pediatric patients under the pressure of nonpharmaceutical interventions for COVID‐19 in Beijing, China

**DOI:** 10.1002/jmv.28411

**Published:** 2022-12-22

**Authors:** Ming‐Li Jiang, Yan‐Peng Xu, Hui Wu, Ru‐Nan Zhu, Yu Sun, Dong‐Mei Chen, Fang Wang, Yu‐Tong Zhou, Qi Guo, Aiping Wu, Yuan Qian, Hang‐Yu Zhou, Lin‐Qing Zhao

**Affiliations:** ^1^ Laboratory of Virology, Beijing Key Laboratory of Etiology of Viral Diseases in Children Capital Institute of Pediatrics Beijing China; ^2^ Graduate School of Peking Union Medical College Beijing China; ^3^ Institute of Systems Medicine Chinese Academy of Medical Sciences and Peking Union Medical College Beijing China; ^4^ Suzhou Institute of Systems Medicine Suzhou China

**Keywords:** endemic infection, epidemiology, evolution, genetic variability, respiratory syncytial virus, virus classification

## Abstract

A series of nonpharmaceutical interventions (NPIs) was launched in Beijing, China, on January 24, 2020, to control coronavirus disease 2019. To reveal the roles of NPIs on the respiratory syncytial virus (RSV), respiratory specimens collected from children with acute respiratory tract infection between July 2017 and Dec 2021 in Beijing were screened by capillary electrophoresis‐based multiplex PCR (CEMP) assay. Specimens positive for RSV were subjected to a polymerase chain reaction (PCR) and genotyped by G gene sequencing and phylogenetic analysis using iqtree v1.6.12. The parallel and fixed (paraFix) mutations were analyzed with the R package sitePath. Clinical data were compared using SPSS 22.0 software. Before NPIs launched, each RSV endemic season started from October/November to February/March of the next year in Beijing. After that, the RSV positive rate abruptly dropped from 31.93% in January to 4.39% in February 2020; then, a dormant state with RSV positive rates ≤1% from March to September, a nearly dormant state in October (2.85%) and November (2.98%) and a delayed endemic season in 2020, and abnormal RSV positive rates remaining at approximately 10% in summer until September 2021 were detected. Finally, an endemic RSV season returned in October 2021. There was a game between Subtypes A and B, and RSV‐A replaced RSV‐B in July 2021 to become the dominant subtype. Six RSV‐A and eight RSV‐B paraFix mutations were identified on G. The percentage of severe pneumonia patients decreased to 40.51% after NPIs launched. NPIs launched in Beijing seriously interfered with the endemic season of RSV.

## 1 INTRODUCTION

Coronavirus disease 2019 (COVID‐19), caused by severe acute respiratory syndrome coronavirus 2 (SARS‐CoV‐2), has rapidly spread since December 2019 and then become a pandemic throughout the world. To control SARS‐CoV‐2 transmission, a series of nonpharmaceutical interventions (NPIs), including social withdrawal, school closures, wearing masks, travel restrictions, personal hygiene improvements, and border closures, have been implemented and are widespread in different countries.^1^ It has been proven that the travel bans in Wuhan delayed the overall epidemic progress in mainland China by 3–5 days and reduced international case transmission by nearly 80%.^2^ In Beijing, China, Level Ⅰ, the highest emergency response to COVID‐19, was launched on January 24, 2020. As the epidemic situation was under control, the NPIs were downgraded to Level Ⅱ on April 30, 2020. Restrictions on public places were gradually lifted, and schools resumed. On June 6, 2020, the NPIs were adjusted to Level III. Due to the outbreak of SARS‐CoV‐2 in the seafood market, the level was upgraded to II from June 17 to July 20, 2020. Then, the level was maintained in III. Wearing masks in public was mandatory under Levels II and III in Beijing. All these strict prevention and control measures effectively prevented the spread of SARS‐CoV‐2.^3^

These NPI measures have also affected the community transmission of endemic diseases and the seasonal circulation patterns of other respiratory viruses,^4^ including influenza viruses (Flu),^5^ respiratory syncytial virus (RSV),^6^ rhinovirus (Rh),^7^ human metapneumovirus (hMPV),^8^ and adenovirus (AdV).^9^ Therefore, it is crucial for all countries to establish sound monitoring systems to prevent and control regional infectious diseases.

RSV is the most important viral cause of acute respiratory tract infections (ARTI) in children and is a major pathogen leading to infant death worldwide.^10^ According to epidemiological data in 2015, the number of ARTIs caused by RSV infection was approximately 33 million globally, resulting in approximately 3.2 million hospitalizations. Among children under 6 months of age, there were 1.4 million hospitalizations for RSV infection and approximately 27 300 child deaths.^11^ RSV has only one serotype, divided into Subtypes A and B, and then into a variety of genotypes based on the gene sequences of the Hypervariable Region 2 (HVR2) of glycoprotein G. In previous studies, the dominant RSV subgroups changed from year to year with a shifting pattern.^12^ The dominant genotype of Subtype A was altered from NA1 to ON1, an emerging genotype reported in 2012 and becoming the dominant genotype since 2015,^13^, ^14^, ^15^, ^16^ while the dominant genotype of Subtype B in recent years remained BA9, which was first reported in China in 2006.^10^, ^17^ The periodic alternating epidemics of Subtypes A and B and the emerging genotypes of ON1 and BA9, which were also reported in Kuwait, Thailand, South Africa, Spain, and the United States, and two novel RSV ON1.1 variants reported in Taiwan that delayed the 2020–2021 RSV outbreak revealed the accumulation of evolutionary locus pressure.^16^, ^18^, ^19^, ^20^ Therefore, it is of great significance to master the epidemiological characteristics of RSV for the prevention and control of regional infectious diseases.

To investigate the changes in endemic patterns of RSV infection in pediatric patients under the pressure of NPIs of COVID‐19 in Beijing, launched on January 24, 2020, clinical specimens from children with ARTI in Beijing from July 2017 to December 2021 were retrospectively analyzed for epidemic characteristics and demographic and clinical features of RSV infection, focused on the data comparison before and after NPIs launched.

## 2 MATERIALS AND METHODS

### 2.1 Clinical specimens

Clinical respiratory specimens were collected from children with ARTI visiting the affiliated Children's Hospital, Capital Institute of Pediatrics (Beijing) from July 1, 2017, to December 31, 2021, for respiratory pathogen screening using a capillary electrophoresis‐based multiplex PCR (CEMP)‐compatible assay‐Respiratory Pathogen Multiplex Detection Kit (Ningbo HEALTH Gene Technologies Ltd.).^21^ The types of specimens included throat swabs, nasopharyngeal swabs, nasopharyngeal aspirates, and bronchoalveolar lavage fluids. Specimens were immediately sent to the laboratory. Upon arrival at the laboratory, each clinical specimen was handled in a Class II biosafety cabinet, processed immediately using 2.5 ml of the viral transport medium (Yocon Biotechnology Co., Ltd.), and then centrifuged (500*g*, 10 min). Partial supernatant was used for viral nucleic acid extraction, and the remaining supernatant was stored at −80°C for future use.

### 2.2 Nucleic acid extraction

Total nucleic acid (DNA and RNA) was extracted from 140 µl supernatant of each collected specimen using the QIAamp MinElute Virus Spin Kit (Qiagen GmbH) according to the manufacturer's instructions.

### 2.3 CEMP assay for multiple pathogen screening

According to the manufacturer's instructions in the CEMP Assay Kit for multiplex polymerase chain reactions (PCRs), deoxynucleoside triphosphates (dNTPs), MgCl_2_, and buffer were included. Nucleic acid extracts from clinical specimens were amplified and then subjected to capillary electrophoresis on a GeXP capillary electrophoresis system (Sciex). Signals of the 15 labeled PCR products were measured by fluorescence and separated into 14 kinds of size fragments, as well as human DNA and human RNA: Flu A 105 nt (2009H1N1 163.3 nt, and H3N2 244.9 nt), Flu B 212.7 nt, AdV 110.2/113.9 nt (representing different subtypes), human bocavirus (HBoV) 121.6 nt, Rh 129.6 nt, human parainfluenza virus 181.6 nt, chlamydia (Ch) genus 190.5 nt, hMPV 202.8 nt, *Mycoplasma pneumoniae* (Mp) 217 nt, human coronavirus (HCoV) 265.1 nt, and RSV 280.3 nt.^21^

### 2.4 Reverse transcription and PCR for RSV subtyping

The extracted nucleic acid of RSV‐positive specimens was used as a template to synthesize complementary DNA (cDNA) by a conventional two‐step reverse transcription reaction with random primers according to the manufacturer's instructions. The moloney murine leukemia virus (M‐MLV) reverse transcriptase (200 U/μl) was used (S28025‐021; Invitrogen). Ribonuclease inhibitor (50 U/μl, Q069; TransGen Biotech) and dNTP (10 mM, C068; TransGen Biotech) were added separately to mix.

For RSV subtyping, cDNA obtained by reverse transcription was used as the PCR template. Forward (P4: 5′‐TGGGACACTCTTAATCAT‐3′) and reverse primers (P5: 5′‐TGATTCCAAGCTGAGGAT3′, P6: 5′‐GTTGTATGGTGTGTTTC‐3′) were used.^10^ The reactions were performed in a 25 µl final volume mixture according to the kits 2×EasyTaq® PCR SuperMix and manufacturer's instructions (AS111‐01; TransGen Biotech). The amplified products of 250 bp for RSV‐A and 341 bp for RSV‐B were visualized by electrophoresis in 2% agarose gels stained with ethidium bromide.

To amplify the full‐length RSV G gene for phylogenetic analysis, nested PCR was used. Primers S4298 (5′‐TGGCCYTAYTTTACACTAATAC‐3′) and F164 (5′‐GTTATGACACTGGTATACCAAC‐3′) in the first round produced a 1489 bp fragment. Primers S4373 (5′‐ATCTCCATCATGATTGCAAT‐3′) and F5763 (5′‐ATAGCCTTTGCTAACTGCAC‐3′) were used in the second round, producing a 1390 bp fragment.^16^ The amplified products were sent to SinoGenoMax, for sequencing.

### 2.5 Phylogenetic analysis

For all the sequences of RSV Subtypes A and B, CD‐hit was introduced to trim the sequences with high similarity (identity >97.5%).^22^ Then, iqtree v1.6.12 was used to construct maximum likelihood phylogenetic trees.^23^ The replacement model was selected automatically by iqtree according to the Bayesian information criterion, and 1000 ultrafast bootstraps were applied. All phylogenetic trees were visualized by ggtree v3.5.1.^24^ The GenBank accession numbers of the reference sequences used in this study are listed in Supporting Information: Table S1.

### 2.6 Detection of parallel and fixed mutations

When a mutation has been fixed in a population and occurs independently in a new strain many times, reducing the likelihood that the mutation was caused by epistatic effects, parallel and fixed (paraFix) mutations occurred. The detection of paraFix mutations was performed with the R package sitePath.^25^ Briefly, the phylogenetic tree and multisequence alignment files constructed by iqtree v1.6.12 as mentioned above based on all sequences of RSV Subtypes A and B, respectively, were sent to sitePath. Then, the paraFix mutations were detected with the recommended default parameter.

### 2.7 Clinical data collection and definition of disease severity

To analyze the clinical features of RSV infection, hospitalized children with a single RSV infection and detailed clinical data were enrolled. Clinical data, such as age, sex, oxygen saturation, respiratory rate (RR), body temperature, and chest radiography or computerized tomography (CT), were collected. The severity was evaluated according to the “Guidelines for the Diagnosis and Treatment of Community‐acquired Pneumonia in Children (2019 edition)” issued by the “China National Health Commission.” Severe community‐acquired pneumonia cases were defined as meeting any of the following criteria: (1) poor general condition, (2) disturbance of consciousness, (3) hypoxemia: cyanosis, rapid respiration, RR ≥ 70 breaths/min (infant), RR ≥ 50 breaths/min (1‐year‐old or older), assisted breathing (groaning, nasal fanning, trisplegia), intermittent apnea, oxygen saturation <92%, (4) hyperthermia, persistent hyperthermia for more than 5 days, (5) dehydration or food refusal, and (6) chest radiograph or CT ≥ 2/3 side lung infiltration, lobar lung infiltration, pleural effusion, pneumothorax, atelectasis, lung necrosis, and lung abscess.

### 2.8 Statistical analysis

January 24, 2020 was set as the boundary before and after the NPIs launched in Beijing. SPSS Statistics (version 22.0; IBM) software was used to process the data. In the descriptive analysis, measurement data with a normal distribution are presented as $\bar{x}$ ± s, while those with a skewed distribution are presented as the median and interquartile range. *χ* ^2^ and rank sum tests were used for statistical analysis. Statistical significance was set at *p* < 0.05.

## 3 RESULTS

### 3.1 Pathogen screening results of the CEMP assay

From July 1, 2017 to December 31, 2021, a total of 10 588 specimens from children with ARTI were collected, and 9198 were finally included after excluding those with short‐term repeated testing and incomplete common clinical information. There were 5400 males and 3798 females, with a mean age of 3.229 (0.001–17.855) years. The positive rates of RSV were 13.56% (1247/9198), hMPV, 2.76% (254/9198), AdV, 3.53% (325/9198), Rh, 16.46% (1514/9198), HBoV, 6.87% (632/9198), HCoV, 0.91% (84/9198), Flu A, 1.64% (151/9198) and Flu B, 0.87% (80/9198), and PIV, 8.12% (747/9198). The positive rates of Mp and Ch were 11.82% (1088/9198) and 0.57% (52/9198), respectively.

**Figure 1:**
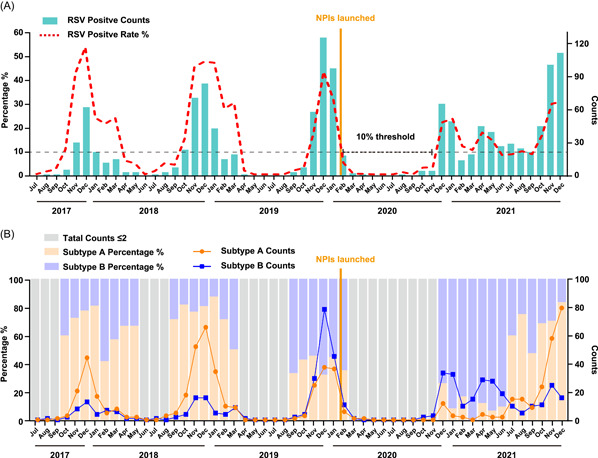
Monthly distribution of RSV‐positive specimens in children with ARTIs in Beijing from Jul 2017 to December 2021. (A) Monthly distribution of RSV‐positive specimens. The numbers of RSV‐positive specimens are shown in the green column diagram, and the positive rates of RSV are shown in the red broken line graph. (B) Monthly distribution of RSV Subtype A‐ and B‐positive specimens. The numbers of RSV A‐ and B‐positive specimens are shown in the line graph with yellow for RSV A and blue for RSV B, respectively, and the proportions of RSV A and B are shown in the column diagram with yellow for RSV A and blue for RSV B, respectively. ARTI, acute respiratory tract infection; RSV, respiratory syncytial virus.

By using January 24, 2020, as the dividing line (Figure 1), when Level I, the highest emergency response to COVID‐19, was launched, 3338 specimens were tested by CEMP assay from July 2017 to January 2020, 19.95% (666/3338) were positive for RSV, 5860 specimens were tested from February 2020 to December 2021, and 9.91% (581/5,860) were positive for RSV. Usually, 10% RSV positive rates were set as the threshold of an endemic season.^16^ From 2017 to 2019, each endemic season started in October or November and ended in the next year's February or March (Figure 1A), lasting for approximately 5–6 months. However, the RSV positive rates decreased abruptly from 31.93% in January 2020 to 4.39% in February 2020. Then, the monthly positive rates of RSV increased quickly to 22.34% in December 2020 and 23.71% in January 2021 and then decreased to 12.03% in February and 10.40% in March 2021. However, the positive rates of RSV increased again in April (17.80%, 42/236) and May 2021 (14.45%, 37/256) and then abnormally stayed at approximately 10% during the summer from June to September 2021, which was followed by a low endemic season of RSV from October 2021.

There were 890 cases among 1247 positive for RSV with a single RSV pathogen, including 521 males and 369 females, with a median age of 0.60 (0.17–1.88) years. In addition, 357 cases were infected with multiple pathogens, 215 males and 142 females, with a median age of 1.19 (0.42–3.11) years. Among these cases, RSV and Rh coinfection accounted for 31.09% (111/357), RSV and HBoV accounted for 13.17% (47/357), and RSV and AdV accounted for 9.80% (35/357). There were 48 cases of triple infection and 13 cases of quadruple infection or above.

### 3.2 Molecular epidemiology of RSV

By subtyping PCR for 1247 RSV‐positive specimens, subtypes of RSV were identified among 1150 specimens (Figure 1B), in which RSV‐A accounted for 54.35% (625/1150), RSV‐B accounted for 44.00% (506/1150), and coinfection of A and B accounted for 1.65% (19/1150). In the 2017–2018 and 2018–2019 epidemic seasons, RSV‐A was the dominant subtype. Then, RSV‐B became dominant (in December 2019, 67.80%) during the 2019–2020 epidemic season. After a long dormant stage of RSV from March to November 2020, RSV‐B was the first to increase quickly from December 2020 and then remained at a relatively high level until June 2021. Then, RSV‐A has a game with RSV‐B and has gradually become dominant since July 2021, with the proportion of RSV‐B decreasing from 90.48% (June 2021) to 40.00% (July 2021) and RSV‐A increasing from 9.52% (June 2021) to 60.00% (July 2021). Then, the proportion of RSV‐A was up to 75.00% in August 2021 and down to 47.37% in September 2021. Finally, the proportion of RSV‐A was over 68.57% competitively with 31.43% of RSV‐B in October 2021.

### 3.3 Phylogenetic analysis of RSV

**Figure 2:**
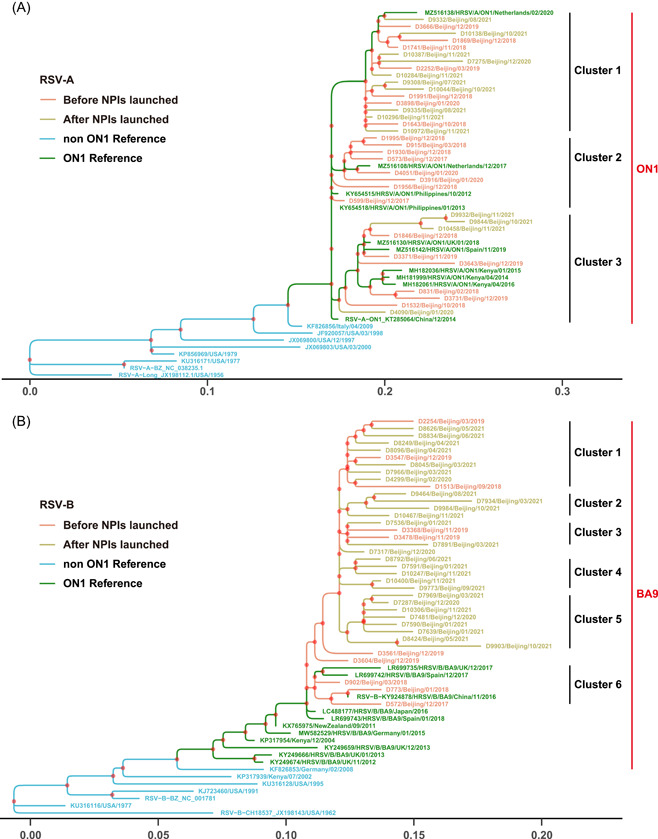
Phylogenetic analysis of RSV G gene sequences in Beijing from 2017 to 2021. (A) ON1 was the predominant prevalent genotype of RSV‐A. Three clusters were divided in phylogenetic trees. (B) BA9 was the predominant prevalent genotype of RSV‐B. Six clusters were divided in phylogenetic trees. These trees were constructed using the maximum likelihood method from 1000 bootstrap samplings by iq‐Tree. Each reference sequence was labeled by GenBank accession number, followed by location and year. Sequences collected in the study were labeled by laboratory number, location and time (m/y). The ON1 and BA9 references are indicated in green, and the other references are indicated in blue. Sequences collected before NPIs launched are shown in red, and sequences collected after NPIs launched are shown in yellow. NPI, nonpharmaceutical interventions; RSV, respiratory syncytial virus.

Full‐length G gene sequences of 302 RSV‐A and 348 RSV‐B were obtained by reverse transcription and nested PCR assays. Before NPIs launched, there were 174 for RSV‐A and 108 for RSV‐B, while after NPIs launched, there were 128 for RSV‐A and 240 for RSV‐B. Phylogenetic analysis revealed that all RSV‐A sequences in the study belonged to the ON1 genotype (Figure 2A), which could be categorized into three clusters, and the sequences were scattered around these clusters. Specifically, D9844, D9932, and D10458, collected after NPIs launched, formed a new branch in cluster 3. All RSV‐B sequences belonged to the BA9 genotype, which could be categorized into six clusters (Figure 2B). Those sequences collected before NPIs launched were more genetically close to those of Spain (GenBank: LR699742) and the United Kingdom (GenBank: LR699735) in 2017. Interestingly, all sequences of RSV B clustered into 2, 4, and 5 were collected after NPIs launched, which were divided into particular lineages.

### 3.4 Potentially adaptive mutations in RSV evolution

Sequences of 61397 (ON1, isolated in Beijing, December 2014) and 86 673 (BA9, isolated in Beijing, August 2017) were used as references before the NPIs launched to analyze amino acid mutants on glycoprotein G. For RSV‐A, 155 and 105 sites and for RSV‐B, 103 and 157 sites were mutated before or after the NPIs launched, respectively. Loci with mutation rates greater than 10% are listed separately and presented in Table 1. In the cytoplasmic, transmembrane, and heparin‐binding domains, Subtype A was relatively conserved, while Subtype B showed mutations at V45I, K207R, and K219T after NPIs launched. In the central conserved domain, the N178G/S mutation appeared in Subtype A, and the N176S mutation appeared in Subtype B.

**Table 1:** Amino acid mutation site, number and rate (%) higher than 10% of G protein in RSV‐A and RSV‐B before or after NPIs launched

	Before NPIs launched			After NPIs launched		
	Site	Numbers	Rate (%)	Site	Numbers	Rate (%)
RSV‐A						
Cytoplasmic domain (1–35 aa)	/	/	/	/	/	/
Transmembrane domains (36–66 aa)	/	/	/	/	/	/
Hypervariable Region Ⅰ (67–163 aa)	/	/	/	A79 V/T	27	20.93
	/	/	/	S111F	28	21.71
	T113I/V	119	69.19	T113I	99	76.74
	V131D	119	69.19	V131D/N	99	76.74
	K134I/T/R	25	14.53	/	/	/
	/	/	/	L142S	28	21.71
Central conserved domain (164–185 aa)	N178G/S	119	69.19	N178G/S	99	76.74
Heparin‐binding domain (186–223 aa)	/	/	/	/	/	/
Hypervariable Region Ⅱ (224–311 aa)	/	/	/	E224A	28	21.71
	V225A	25	14.53	V225A/S	33	25.58
	/	/	/	L226I/P	17	13.18
	L230P/T	171	99.42	L230P/T	127	98.45
	V236I	171	99.42	V236I	128	99.22
	K244R	171	99.42	K244R	125	96.90
	T245A	25	14.53	T245A	21	16.28
	/	/	/	L247P	28	21.71
	H258Q/Y	123	71.51	H258Q	99	76.74
	E262K/G	23	13.37	/	/	/
	H266L	119	69.19	H266L	99	76.74
	/	/	/	Y273H	13	10.08
	L274P	31	18.02	L274P	80	62.02
	F280Y/H/N	171	99.42	F280Y/H	128	99.22
	/	/	/	T282I	28	21.71
	/	/	/	G284S/D	14	10.85
	K286E	165	95.93	K286E	118	91.47
	/	/	/	L289I	25	19.38
	/	/	/	G296S/D	29	22.48
	L298P	30	17.44	L298P/I	48	37.21
	/	/	/	P300Q/S	19	14.73
	Y304H	21	12.21	Y304H	36	27.91
	/	/	/	E308K/V	31	24.03
	/	/	/	L310P/S	41	31.78
	/	/	/	S311P	29	22.48
	/	/	/	L314P/F	31	24.03
	K320T/I/A	171	99.42	K320T/A	128	99.22
RSV‐B						
Cytoplasmic domain (1–35 aa)	/	/	/	/	/	/
Transmembrane domains (36–66 aa)	/	/	/	V45I	35	14.46
Hypervariable Region Ⅰ (67–163 aa)	/	/	/	R98G/K/M	81	33.47
	/	/	/	E125D/K	27	11.16
	/	/	/	T129K/X	26	10.74
	A131T	86	79.63	A131T/X	238	98.35
	T137I	86	79.63	T137I/V	241	99.59
Central conserved domain (164–185 aa)	N176S	11	10.19	N176S	67	27.69
Heparin‐binding domain (186–223 aa)	P201T	107	99.07	P201T	241	99.59
	/	/	/	K207R	25	10.33
	Y213D	105	97.22	Y213D/A	241	99.59
	P217L	103	95.37	P217L	203	83.88
	/	/	/	K219T	25	10.33
	P221L/S	16	14.81	/	/	/
Hypervariable Region Ⅱ (224–311 aa)	G241D/V	107	99.07	G241D/N	240	99.17
	/	/	/	V249A/E	32	13.22
	I252T/M	23	21.30	I252T/M	47	19.42
	T253I/A	11	10.19	/	/	/
	/	/	/	S265P/L	33	13.64
	/	/	/	I268T	26	10.74
	V269A	93	86.11	V269A	153	63.22
	T274A	12	11.11	T274A/V	67	27.69
	P284L/I	105	97.22	P284L	241	99.59
	Y285H	22	20.37	/	/	/
	T288I	105	97.22	T288I	241	99.59
	/	/	/	N294Y/H	54	22.31
	/	/	/	T300I	34	14.05
	P301A/V	106	98.15	P301A/T/V	241	99.59
	I310T/V	20	18.52	/	/	/

Abbreviations: aa, amino acids; NPI, nonpharmaceutical interventions; RSV, respiratory syncytial virus.nonpharmaceutical interventions.

**Figure 3:**
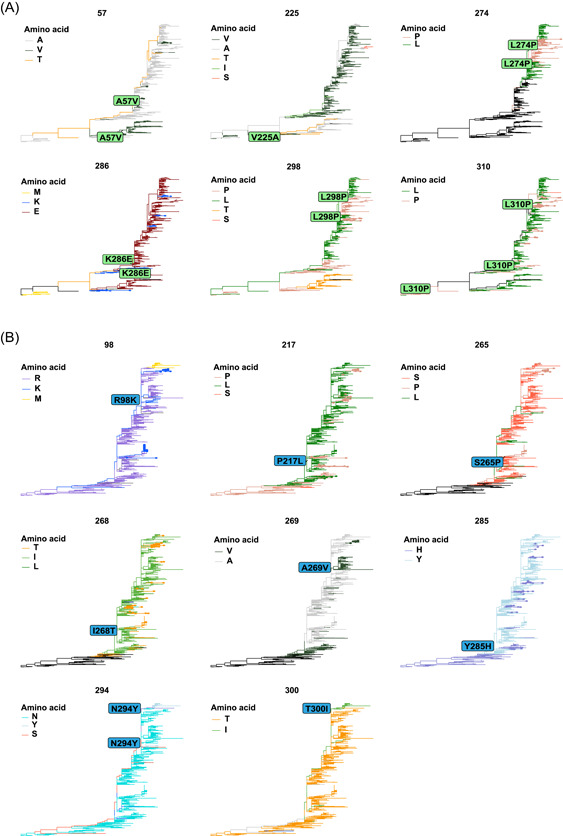
Mutations in RSV G protein in parallel and fixed (paraFix) patterns. The phylogenetic pathways were identified, and fixed mutations were detected along each phylogenetic pathway. paraFix mutations are detected by integrating the fixed and parallel patterns. (A) Six mutations were detected among ON1 sequences. (B) Eight mutations were detected among BA9 sequences. RSV, respiratory syncytial virus.

Sequences of RSV‐A and RSV‐B were aligned and compared with all genotypes in the evolution of viruses. Specific mutations of ON1 in Beijing were T113I, V131D, N178G, H258Q, and H266L. No specific mutation of BA9 was found in Beijing. Furthermore, mutations in parallel and fixed (paraFix) patterns were found by identifying the polymorphism clades in each phylogenetic pathway in global circulation (Figure 3). Among ON1 sequences, A57V, V225A, K286E, and L310P mutations emerged and fixed many times in RSV evolution and recently emerged in sequences in Beijing, while L274P and L298P mutations experienced reverse mutation as “L to P to L.” Among BA9 sequences, R98K, P217L, S265P, I268T, A269V, Y285H, N294Y, and T300I mutations emerged and fixed many times in RSV evolution and recently emerged in sequences in Beijing. Among these mutations, R98M, I268T, and T300I were mutated only after NPIs launched.

### 3.5 Clinical characteristics of RSV‐infected children

**Figure 4:**
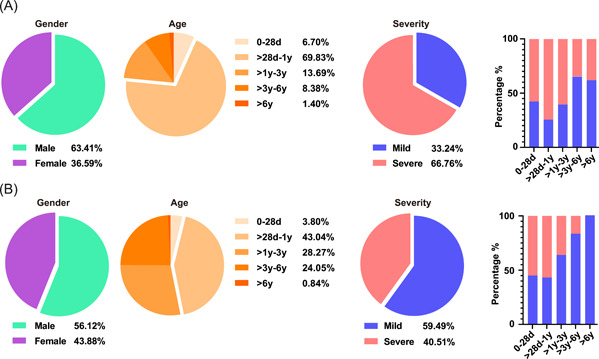
Demographic and clinical symptoms and severity of children who were single positive for RSV in Beijing with ARTIs from July 2017 to January 2020 (A) and from February 2020 to December 2021 (B). The proportions of different sexes, ages and severities of RSV‐positive children are described by the pie chart before (A) and after NPIs launched (B). The proportion of children with mild or severe disease in different age groups is shown in a bar chart. ARTI, acute respiratory tract infection; NPI, nonpharmaceutical interventions; RSV, respiratory syncytial virus.

Among 890 cases with a single RSV infection determined in the study, 595 cases were inpatients with detailed clinical data. There were 360 males and 235 females, with a median age of 0.48 (0.16–1.81) years. The clinical characteristics of RSV‐infected children in the hospital before and after NPIs launched were compared on the basis of sex, age, and severity of pneumonia, the criteria for grouping (Figure 4). In the gender distribution, the proportion of male patients was 63.41% (227/358) from July 2017 to January 2020 and 56.12% (133/237) from February 2020 to December 2021 (*p* = 0.075). In view of the developmental status and growing environment of children, the five groups were gradually distinguished by age. The highest number of RSV infections was observed among children aged 28 days to 1 year (59.16%, 352/595), and the lowest was observed among children aged >6 years (1.18%, 7/595). From before to after NPIs launched, the distribution of RSV decreased from 69.83% (250/358) to 43.04% (102/237) among those aged 28 days to 1 year (*p* < 0.001), while it increased from 13.69% (49/358) to 28.27% (67/237) (*p* < 0.001) and 8.38% (30/358) to 24.05% (57/237) (*p* < 0.001) among children aged >1–3 years and >3–6 years, respectively.

Notably, the percentage of severe pneumonia patients also changed from 66.76% (239/358) to 40.51% (96/237) (*p* < 0.001). Furthermore, we observed changes in sex and age in patients with different severities of pneumonia (Figure 4). In children aged 28 days to 1 year and >1–3 years, the proportions of severe pneumonia patients decreased from 72.80% (182/250) to 55.88% (57/102) (*p* < 0.001) and 61.22% (30/49) to 37.31% (25/67) (*p* = 0.0108). In detail, hospitalization duration, intensive care unit(ICU) admission, and clinical outcome were counted to evaluate the severity of ARTI (Table 2). The average hospitalization duration (6.92 ± 4.23 days vs. 6.16 ± 3.37 days) showed no significant differences, with the proportion of >11 days hospitalization duration decreasing from 15.9% (38/239) to 9.3% (9/96) (*p* = 0.1200) from before to after NPIs launched, while the proportion of ICU admission also dropped from 73.6% (176/239) to 59.4% (57/96) (*p* = 0.0103), and 4.2% (4/96) more children were cured during the hospital stay after NPIs launched.

**Table 2:** Severity classification of RSV‐positive patients in the surveillance period

Period	Before NPIs launched	After NPIs launched	*p* Value
*Hospitalization duration (days)*			
≤6	50.6% (121/239)	43.8% (42/96)	0.2548
6–11	33.5% (80/239)	46.9% (45/96)	0.0218
>11	15.9% (38/239)	9.3% (9/96)	0.1200
*ICU admission*			
Yes	73.6% (176/239)	59.4% (57/96)	0.0103
*Clinical outcome*			
Cured	0.0% (0/239)	4.2% (4/96)	0.0015
Improved	99.2% (237/239)	95.8% (92/96)	0.0377
Dead	0.4% (1/239)	0.0% (0/96)	0.5256
Others	0.4% (1/239)	0.0% (0/96)	0.5256

Abbreviations: ICU, intensive care unit; NPI, nonpharmaceutical interventions; RSV, respiratory syncytial virus.

## 4 DISCUSSION

In the study, it was found that the positive rate of RSV infection in children abruptly dropped from 31.93% in January 2020 to 4.39% in February 2020 under the Level I NPIs. The highest emergency response to COVID‐19 was launched in Beijing on January 24, 2020, and then remained in a truly dormant state in the next 7 months from March to September 2020, with monthly positive rates of RSV less than 1% and a nearly dormant state in October (2.85%) and November (2.98%) under the Level II or III NPIs. It has been confirmed that droplets and aerosols are common ways of transmitting respiratory viruses, such as SARS‐CoV‐2 and RSV, while direct contact is another common route of RSV transmission. Isaacs's et al.^26^ demonstrated that the use of alcohol for hand disinfection significantly reduced the nosocomial infection rate of RSV. British scholars also observed the destructive effect of alcohol compounds on RSV through electron microscopy.^27^ In addition, targeted protective measures and standardized epidemic prevention and control procedures in hospitals and other health institutions may also be one of the factors to reduce nosocomial RSV infection. Under NPIs, wearing a mask can effectively inhibit the spread of the virus, and hand washing and contact protection are important measures to prevent the transmission of the virus. Therefore, it can be explained that there are corresponding changes in the regional epidemic trend of RSV under the pressure of NPIs to SARS‐CoV‐2.^28^, ^29^, ^30^

However, the monthly positive rates of RSV then increased quickly to 22.34% in December 2020 and 23.71% in January 2021 with the relaxation of NPIs in Beijing and then decreased to 12.03% in February and 10.40% in March 2021. Abnormally, the positive rates of RSV increased again in April (17.80%, 42/236) and May 2021 (14.45%, 37/256) and then stayed at approximately 10% during the summer from June to September 2021, which was followed by a normal epidemic season of RSV from October 2021, with the highest positive rate in December 2021 (30.95%, 104/336), although far below the corresponding rates (54.21%, 48.15%, and 43.82%) in 2017, 2018, and 2019. This is consistent with the delayed surge of RSV in other areas.^20^, ^31^, ^32^ A prediction model based on multicenter data suggests that the infection rates of RSV and other associated respiratory viruses will rebound with the relaxation of control strategies, and healthcare systems need to be prepared for outbreaks of non‐SARS‐CoV‐2 infections in the coming years.^33^ At present, the global epidemic of COVID‐19 has not been effectively controlled, which will continue to have an impact on the epidemic characteristics of RSV.

From 2017 to 2021, the alternating predominant‐subtyping epidemic model in Beijing of RSV was AABBA (Figure 2B). In 2021, under NPIs such as “restricted access to Beijing” and “home monitoring,” RSV in Beijing has also completed the replacement of dominant epidemics of Subtypes B to A. Therefore, the replacement of subtypes in certain areas is not necessarily caused by the importation of dominant strains from other countries.^34^ What is relatively special is that we definitely caught the game between Subtypes A and B, and finally, RSV‐B was surpassed by RSV‐A in July 2021, with the proportion of RSV‐B decreasing from 90.48% (June 2021) to 40.00% (July 2021) and RSV‐A increased from 9.52% (June 2021) to 60.00% (July 2021). Normally, the replacement of subtypes was monitored at the beginning of a new epidemic season because no positive specimen was detected, usually in the summer season. Therefore, the abnormal RSV prevalence in the summer of 2021 gives us a clearer understanding of the occurrence of subtype switching in summer. Our data are up to December 2021, and we can observe that Subtype A gradually retransformed into the dominant epidemic strain, which may further lead to changes in the severity of disease caused by virus infection. Therefore, long‐term continuous surveillance can further reflect the profound impact of COVID‐19 on changes in RSV epidemiological characteristics.

Full‐length sequencing of the G protein was performed in our experiment. We referenced the earlier ON1 and BA1 sequences as templates to analyze the changes in the overall mutation rate of G protein amino acid sites. We found that the overall mutation number of the Subtype A virus decreased from 155 to 105 after NPIs launched, while the mutation number of the Subtype B virus increased from 103 to 157, showing the completely opposite trend. This is a very interesting result. We speculate that this may be related to the alternate epidemic of Subtypes A and B to a certain extent. It was reported that the pre‐existing antibodies in the population have great pressure on the virus variation.^35^ According to the research results, we assumed that the NPIs of COVID‐19 were another kind of pressure on the RSV epidemic mode. Under the dual pressure, strains of Subtype B, the predominant subtype in the epidemic season of 2018–2019 and 2019–2020, tried to escape these pressures through more mutations and were eventually overthrown. In contrast, there were fewer pre‐existing antibodies of Subtype A in the population in the epidemic seasons of 2018–2019 and 2019–2020. Although there were fewer mutations among strains of Subtype A, the main subtype was transformed from B to A. More immunological tests should be performed to test the hypothesis.

For the specific amino acid sites, such as T113I, V131D, L230P/T, V236I, K244R, F280Y, K286E, and K320T in RSV‐A and A131T, T137I, P201T, Y213D, P217L, G241D, V269A, P284L, T288I, and P301A in RSV‐B, the replacement of the previous loci was basically completed. Nevertheless, similar important mutation sites have been reported in the other provinces of China and other countries, which also indicates that samples from different regions may vary to a certain extent. To further verify the adaptability of these mutations, paraFix analysis was performed. The paraFix mutations occur when a mutated site has been fixed in a population, reducing the likelihood caused by epistatic effects. This method has been validated in the presence of important mutant strains of SARS‐CoV‐2.^25^ In our data, three paraFix mutations were “L” to “P,” as L274P, L298P, and L310P were identified in the RSV‐A subtype, which indicated that these sites might be the key amino acids on the G protein. In addition, the paraFix mutations were distributed mainly in HVR2 in both RSV‐A and RSV‐B subtypes. The prevailing genotypes of RSV also exhibit 72‐nucleotide duplication in HVR2. Therefore, paraFix mutations might become the basis for RSV typing. Furthermore, serological analysis of clinical samples, statistics of changes in patients' immune levels, and multiomics sequencing technology may further reveal the causes of changes in the mutation rates and mutation sites of the RSV G protein. Reverse genetics techniques should be used to construct mutant viruses to verify the importance of these paraFix sites to RSV and further determine the specific mechanisms by which these mutations affect the virus life cycle.

In addition, clinical characteristics were also influenced by the NPIs. The most prominent age group of RSV infection was 28‐days to 1‐year‐old children. After NPIs launched, the distribution of RSV decreased from 69.83% to 43.04%. This may be due to the decrease in RSV exposure caused by various NPIs during the period of COVID‐19 in Beijing and the accumulation of RSV‐susceptible people resulting in the delay or even outbreak of the epidemic. According to the data on hospitalization duration, ICU admission, and clinical outcome, we found that the severity of ARTI caused by RSV decreased significantly, which was similar to the results in Shanghai, China.^31^ However, some studies have shown that the clinical symptoms of respiratory tract infections caused by RSV are more serious during the COVID‐19 pandemic.^28^, ^36^ Although reasonable speculation, these differences might be associated with the discrepant NPIs between China and the USA. In Beijing, children would be more active in medical treatment and drug intervention, referring to epidemic prevention and control strategies. However, children in the USA were at risk of coinfection with RSV and other respiratory viruses, such as SARS‐CoV‐2, and their immune systems might be affected and suppressed, thus showing more severe clinical symptoms.

In this study, we summarized the epidemiology and clinical phenotype changes of RSV from 2017 to 2021 in Beijing. The pressure of NPIs of COVID‐19 affected the transmission pattern of RSV, which led to the delay of epidemic seasons in 2020 and the abnormal increase in RSV‐positive rates in the summer of 2021. The replacement of subtypes was surveilled from B to A in the summer first. Furthermore, the paraFix mutations of important amino acid sites are of great significance to the development of drugs and vaccines under the new situation. The age distribution of RSV infection was changed due to the NPIs of COVID‐19, and the clinical data showed the lighter phenotype of ARTI. The surveillance of respiratory‐associated viruses should be maintained under the COVID‐19 strategy to avoid unexpected outbreaks of other diseases.

## AUTHOR CONTRIBUTIONS

Ming‐Li Jiang, Yan‐Peng Xu, Hui Wu, and Hang‐Yu Zhou performed the experiments. Lin‐Qing Zhao conceived and designed the experiments. Ru‐Nan Zhu, Yu Sun, Dong‐Mei Chen, Fang Wang, Yu‐Tong Zhou, Qi Guo, Aiping Wu, and Yuan Qian contributed reagents/materials/analysis tools. Yan‐Peng Xu and Lin‐Qing Zhao wrote the paper. Aiping Wu, Yuan Qian, Hang‐Yu Zhou, and Lin‐Qing Zhao reviewed the manuscript. Lin‐Qing Zhao is the critical reviewer for this manuscript and made the final decision on the manuscript. All authors have read and approved the final manuscript.

## CONFLICT OF INTEREST

The authors declare no conflict of interest.

## ETHICS STATEMENT

The original study was approved by the Ethics Committee of the Capital Institute of Pediatrics (Approval Number: SHERLLM2022027) and was a retrospective and prospective study. The ethical committee voted that written informed consent was not required for respiratory syncytial virus RNA‐positive specimens collected retrospectively.

## Supporting information

Supplementary information.

## Data Availability

Data sharing is not applicable to this article as no new data were created or analyzed in this study.
